# An Analysis of the Racial Disparities Among Cervical Cancer Patients Treated at an Academic Medical Center in the Southeastern United States

**DOI:** 10.7759/cureus.13296

**Published:** 2021-02-12

**Authors:** Toms Vengaloor Thomas, Shivanthidevi Gandhi, Eldrin Bhanat, Kati Krishna, William Robinson, Mildred Ridgway, Anu Abraham, Srinivasan Vijayakumar, Satya Packianathan

**Affiliations:** 1 Radiation Oncology, University of Mississippi Medical Center, Jackson, USA; 2 Orthopaedic Surgery, University of Mississippi Medical Center, Jackson, USA; 3 Obstetrics and Gynecology, University of Mississippi Medical Center, Jackson, USA; 4 Pathology, Universtiy of Mississippi Medical Center, Jackson, USA

**Keywords:** racial disparities, cervical cancer, cancer, oncology, radiation, radiation oncology, disparities, cervical oncology, gynaecology and obstetrics, gynae oncology

## Abstract

Objective

The purpose of this study was to identify racial disparities in treatment outcomes, if any, among patients with carcinoma of the cervix treated at a tertiary care institution in the state of Mississippi.

Methods

A retrospective review of patients with carcinoma of the cervix treated in the Department of Radiation Oncology at our institution between 2010 and 2018 was performed. Data regarding demographics, disease stage, treatments administered, and follow-up were collected. Patient outcomes, including median survival and overall survival, were analyzed using the Kaplan-Meier method. All analyses were performed using SPSS Statistics version 24 (IBM, Armonk, NY).

Results

Between January 2010 and December 2018, a total of 165 patients with carcinoma of the cervix were treated at our institution. We had a significantly higher proportion of African American (AA) compared to Caucasian American (CA) patients (59.4 vs. 36.4%; p=0.03). There was a significant difference in the disease stage at the time of presentation between AA and CA in that compared to AA women, a higher number of CA patients presented with locally advanced disease [Federation of Gynecology and Obstetrics (FIGO) stages IB2 to IVA] (78.6 vs. 86.7%; p<0.001). However, a higher number of AA patients presented with metastatic disease at diagnosis compared to CA women (13.3 vs. 8.3%; p<0.001).

Regarding their treatment, 157 (95.2%) underwent definitive chemoradiotherapy, while three (1.8%) had definitive surgery followed by adjuvant radiation or chemoradiation, depending on the risk factors identified operatively. The treatment details of five patients were not available. The median follow-up and the median survival of the entire cohort were 16 months and 79 months, respectively. In our cohort, there was no significant difference in overall survival between AA and CA patients at either three years (80 vs. 68%; p=0.883) or five years (77 vs. 68%; p=0.883). As expected, patients with locally advanced disease showed a significantly better median survival of 79 months compared to only 11 months for those with metastatic disease at their presentation (p<0.001).

Conclusions

Our study revealed that more AA women presented with metastatic disease compared to CA women. However, our analysis did not identify any racial disparities in the prognosis of the entire cohort.

## Introduction

Cervical cancer is the third most prevalent gynecological malignancy in the United States (US). The incidence of cervical cancer is 9.2 and 7.1 per 100,000 people among African Americans (AA) and Caucasian Americans (CA), respectively, while the mortality rate associated with the condition is disproportionately higher among AA (3.6 vs. 2.1/100,000) [1]. Another recent analysis of trends in racial and regional disparities in cervical cancer outcomes also reported that the black race and the southern region of the US are associated with a higher incidence of cervical cancer [2]. AA women tend to present at a higher stage of disease compared to CA women [3], and many authors have also reported on racial disparity in the treatment of cervical cancer between AA and CA patients, resulting in differences in survival outcome [4,5].

In 2019, the overall incidence of cervical cancer in the state of Mississippi was 140/100,000 [1]. Horner et al. examined the geographical distribution of cervical cancer cases and reported that the Mississippi River valley had a high incidence of and mortality from this disease [6,7]. A study of cervical cancer screening among women in the Mississippi Delta region observed that while 85.5% (95% CI: 84.3-86.6%) of eligible women had undergone a Papanicolaou (Pap) test, Pap testing rates were lower among older (≥65 years) Delta women or women who had not visited a doctor within the past year, compared to their counterparts elsewhere [8]. Although cervical cancer mortality was similar in the Mississippi Delta compared to the rest of the US, the rate had declined more rapidly elsewhere than in the Mississippi Delta. Besides, cervical cancer mortality was found to be higher for black women in both the Delta and the US as a whole. Cervical cancer mortality was also noted to be higher among both rural white and urban black women in the Delta compared to their counterparts elsewhere [9].

As Mississippi’s only academic medical center and safety net hospital, a significant number of patients with cervical cancer are treated at the University of Mississippi Medical Center (UMMC). Our purpose in this study was to assess racial disparities, if any, in disease incidence and outcomes among cervical cancer patients treated at our institution.

This work was previously presented as a poster at the American Radium Society meeting, 2020, and was published in the abstract form in the International Journal of Radiation Oncology, Biology, Physics on October 1, 2020 [10].

## Materials and methods

A retrospective review of patients with carcinoma of the cervix was undertaken to evaluate the racial disparities in the presentation and outcomes. All patients had been treated in the Department of Radiation Oncology at our institution between 2010 and 2018. The institutional review board (IRB) of the UMMC approved all the investigations. The need to obtain a written consent was waived due to the retrospective nature of the study. Data of patients diagnosed between 2010 and 2018 were collected by a review of patient charts from the Cervical Cancer Database of UMMC. Research Electronic Data Capture (REDCap), a browser-based database tool, was used to gather and store patient information in password-protected computers.

Data regarding demographics, stage, treatment administered, and follow-up was extracted from the medical records. The institutional cancer registry provided the data on follow-up and vital statistics of the patients.

Patient outcomes, including median survival and overall survival, were evaluated using the Kaplan-Meier method. All analyses were performed using SPSS Statistics version 24 (IBM, Armonk, NY).

## Results

We identified 165 patients with carcinoma of the cervix who were treated at UMMC between January 2010 and December 2018. There was a significantly higher proportion of AA compared to CA patients (59.4 vs. 36.4%; p=0.03; Table 1). There was a significant difference in the disease stage at the time of presentation between AA and CA in that compared to AA women, a higher number of CA patients presented with locally advanced disease [Federation of Gynecology and Obstetrics (FIGO) stages IB2 to IVA] (78.6 vs. 86.7%; p<0.001). However, a higher proportion of AA women presented with metastatic disease at diagnosis (13.3 vs. 8.3%; p<0.001) compared to CA women. With regard to their treatment, 157 (95.2%) underwent definitive chemoradiotherapy, while three (1.8%) had definitive surgery followed by adjuvant radiation or chemoradiation depending on the risk factors identified operatively. The treatment details for five patients were not available.

**Table 1:** Demographic distribution of patients The median follow-up period for the entire cohort was 16 months

Race	N (%)	P-value
Blacks	98 (59.4%)	0.03
Whites	60 (36.4%)	
Others	7 (4.2%)	

**Figure 1:**
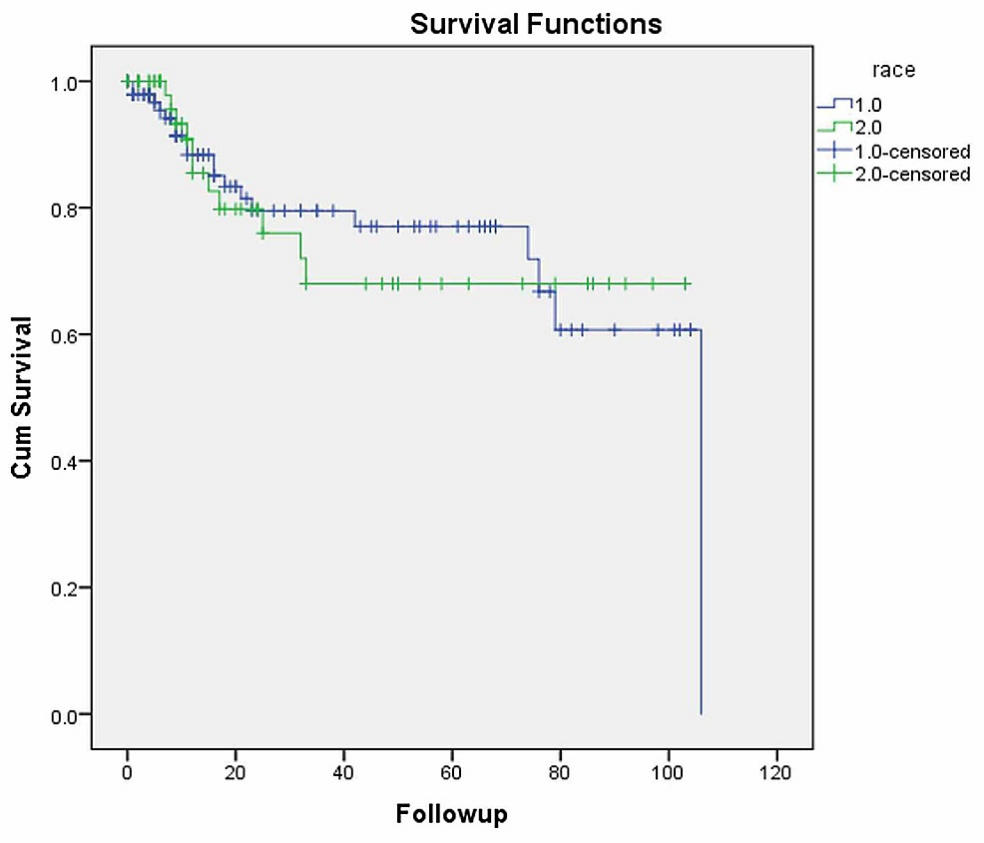
Kaplan-Meier overall survival for cervical carcinoma patients by race

The median follow-up and the median survival of the entire cohort were 16 and 79 months, respectively. In our cohort, there was no significant difference in overall survival between AA and CA patients (Figure 1) at three (80 vs. 68%; p=0.883) or five years (77 vs. 68%; p=0.883). As expected, patients with only locally advanced disease showed a significantly improved median survival of 79 months compared to 11 months for those with metastatic disease at presentation (p<0.001).

## Discussion

Racial disparities in the incidence and prevalence of cervical cancer

Cervical cancer is the third most prevalent gynecological cancer in the US. The estimated incidence and mortality of cervical cancer in 2019 was 13,170 and 4,250, respectively [1]. The incidence and mortality of this disease had been gradually decreasing since the mid-twentieth century, secondary to the acceptance and widespread use of Pap smear screening [11]. Indeed, the incidence has been decreasing by about 0.2% per year and mortality has been decreasing by 0.7% per year [11,12]. 

The national incidence rate of cervical cancer is 9.2 and 7.1 per 100,000 women among AA and CA, respectively, while the mortality is disproportionately higher among AA (3.6 vs. 2.1/100,000 women) [1]. Another report examining recent trends in racial and regional disparities in cervical cancer reported that the black race and southern region of the US have an association with a higher incidence of cervical cancer [2]. The reasons for the increased incidence of cervical cancer among AA women are likely multi-factorial. For instance, although routine vaccination of adolescent girls for the human papillomavirus (HPV) vaccine is recommended [13,14], studies by Niccolai et al. and Widdice et al. have shown that adolescent black girls are less likely to complete their course of vaccination as scheduled [15,16]. In addition, recent reports suggest that the rate of cervical cancer screening has improved among AA women and racial disparities are not as evident in screening processes currently [17], even though significant disparities existed previously [18]. Our patient cohort had a higher number of AA patients, but it is likely due to our general patient population. We currently do not have the data to do an analysis of racial differences in the incidence of cervical cancer.

Racial disparities in the disease stage at presentation

AA women tended to present at a higher stage of disease compared to CA women [3]. Fleming et al. analyzed data from the Maryland Cancer Registry and reported that AA women were more likely to have locally advanced or metastatic disease at diagnosis (p<0.01) [19]. Adams et al. also reported that a higher fraction of AA patients presented with regional or metastatic disease compared to their CA counterparts [20]. In our analysis, we found a significant difference in the stage at the time of presentation between AA and CA. Interestingly, however, compared to AA, a higher fraction of CA patients presented with locally advanced disease (FIGO stages IB2 to IVA) (78.6 vs. 86.7%; p<0.001). However, a higher number of AA women presented with metastatic disease at their diagnosis (13.3 vs. 8.3%; p<0.001) compared to CA. 

Racial disparities in the treatment 

Many authors have reported on the racial disparities in the treatment of cervical cancer between AA and CA patients. Del Carmen et al. have reported that AA women were less likely to receive radical hysterectomy for early-stage disease (stage IA) [4]. A report from the University of Alabama also mentioned that when compared to AA, CA women with early-stage cervical carcinoma were more likely to undergo surgical management (84 vs. 93%; p<0.01) [21]. In addition, AA women were more likely to receive radiation treatment or chemotherapy combined with radiation and were less likely to undergo surgery [19]. Mundt et al. have reported on AA women having a higher likelihood of comorbid conditions, leading to treatment protraction and the inability to complete their brachytherapy boost [22]. Robin et al. noted that AA patients were less likely to receive standard of care chemoradiation treatment [23]. Alimena et al. analyzed information from the National Cancer Database (NCDB) and reported that AA patients were less likely to receive brachytherapy as part of definitive chemoradiotherapy (OR: 0.87, 95% CI: 0.79-0.96; p=0.007), which consequently resulted in a survival detriment [5]. In our patient cohort, all AA patients underwent definitive chemoradiotherapy, while a small fraction of CA patients (5%) underwent surgery followed by adjuvant radiation treatment. This finding is consistent with those of other studies in the literature.

Racial disparities in survival 

Conflicting data exist regarding racial disparities in survival between AA and CA patients. Adams et al., using the South Carolina Cancer Registry, suggested that AA patients have significantly lower overall survival even after being matched stage for stage [20]. A Surveillance, Epidemiology, and End Results (SEER) database analysis also identified the black race and the southern region of the US as manifesting higher mortality from cervical cancer [2]. Mayadev et al. reported that AA patients had worse cancer-specific survival and overall survival compared to CA based on information from the California Cancer Registry [24]. On the other hand, Weragoda et al. retrospectively analyzed cervical cancer patients from two large hospitals in the Southeastern US and reported that there were no racial differences in five-year survival between the races on multivariate analysis [25]. Moreover, an analysis by Mundt et al. did not identify race as a significant factor affecting overall survival in cervical cancer patients [22]. Grigsby et al. reviewed 922 cervical cancer patients from the Mallinckrodt Institute of Radiology and reported an absence of racial disparities in their survival outcomes [26]. Similarly, in our retrospective analysis, we identified no significant difference in overall survival between AA and CA patients at three years (80 vs. 68%; p=0.883) or at five years (77 vs. 68%; p=0.883), although it was a small cohort of patients.

Possible reasons for racial disparities in cervical cancer

There have been multiple reports detailing the reasons for racial disparities in incidence and mortality. Deshmukh et al. have proposed the possibility of racial differences in the biology of the disease as a reason for disparities in the outcomes [27]. Nonzee et al. reported that delays in cancer care among low-income patients, despite access to care, stemmed from a lack of knowledge about the availability of resources, denial or fear, competing obligations, and embarrassment, potentially leading to diminished outcomes [28].

Study limitations and future research directions

The retrospective nature of this analysis and the limited number of patients involved are two of its significant limitations. Confounding variables, like medical comorbidities, which may have provided the witnessed survival differences, could have influenced our study results. A large, prospective study would help to further delineate the details of racial disparities in the incidence, treatment, and prognosis of cervical cancer. Large-scale policy initiatives should be undertaken to improve existing disparities.

## Conclusions

A retrospective review of patients with carcinoma of the cervix treated over the past eight years at a major academic medical center revealed that more AA women presented with metastatic disease compared to CA women. However, our analysis did not reveal significant racial disparities regarding the prognosis of the entire cohort. This study suggests that when provided equal access to care, patients are more likely to have a similar prognosis despite racial variances. However, further studies are needed to validate this hypothesis.
